# Large uniaxial magnetostriction with sign inversion at the first order phase transition in the nanolaminated Mn_2_GaC MAX phase

**DOI:** 10.1038/s41598-018-20903-2

**Published:** 2018-02-08

**Authors:** Iuliia P. Novoselova, Andrejs Petruhins, Ulf Wiedwald, Árni Sigurdur Ingason, Thomas Hase, Fridrik Magnus, Vassilios Kapaklis, Justinas Palisaitis, Marina Spasova, Michael Farle, Johanna Rosen, Ruslan Salikhov

**Affiliations:** 1 Faculty of Physics and Center for Nanointegration (CENIDE), University of Duisburg-Essen, 47057 Duisburg, Germany; 2 Thin Film Physics, Department of Physics, Chemistry and Biology (IFM), Linköping University, SE-581 83 Linköping, Sweden; 3 National University of Science and Technology «MISIS», 119049 Moscow, Russian Federation; 4 Grein Research ehf. Dunhaga 5, Reykjavik, Iceland; 5 Department of Physics, University of Warwick, Coventry, CV4 7AL UK; 6 Science Institute, University of Iceland, Dunhaga 3, IS-107 Reykjavik, Iceland; 7 Division of Materials Physics, Department of Physics and Astronomy, Uppsala University, Box 516, SE-75121 Uppsala, Sweden; 8 Center for Functionalized Magnetic Materials (FunMagMa), Immanuel Kant Baltic Federal University, Kaliningrad, Russian Federation; 9 Zavoisky Physical-Technical Institute, Russian Academy of Sciences, 420029 Kazan, Russian Federation

## Abstract

In 2013, a new class of inherently nanolaminated magnetic materials, the so called magnetic MAX phases, was discovered. Following predictive material stability calculations, the hexagonal Mn_2_GaC compound was synthesized as hetero-epitaxial films containing Mn as the exclusive M-element. Recent theoretical and experimental studies suggested a high magnetic ordering temperature and non-collinear antiferromagnetic (AFM) spin states as a result of competitive ferromagnetic and antiferromagnetic exchange interactions. In order to assess the potential for practical applications of Mn_2_GaC, we have studied the temperature-dependent magnetization, and the magnetoresistive, magnetostrictive as well as magnetocaloric properties of the compound. The material exhibits two magnetic phase transitions. The Néel temperature is *T*_*N*_  ~ 507 K, at which the system changes from a collinear AFM state to the paramagnetic state. At *T*_*t*_ = 214 K the material undergoes a first order magnetic phase transition from AFM at higher temperature to a non-collinear AFM spin structure. Both states show large uniaxial *c*-axis magnetostriction of 450 ppm. Remarkably, the magnetostriction changes sign, being compressive (negative) above *T*_*t*_ and tensile (positive) below the *T*_*t*_. The sign change of the magnetostriction is accompanied by a sign change in the magnetoresistance indicating a coupling among the spin, lattice and electrical transport properties.

## Introduction

Inherently nanolaminated *M*_*n*+*1*_*AX*_*n*_ (*n* = 1, 2, 3) compounds attract tremendous interest, since these materials provide the unique anisotropic structural and physical properties important for diverse applications^1,2^. These compounds, collectively known as MAX phases, are composed of an early transition metal (*M*), a *p*-element from the A-group elements (*A*) and *X* being either C or N. MAX phases have a hexagonal structure and belong to the space group P6_3_/mmc with the primitive unit cell given by 8 atoms: 4 *M*, 2 *A* and 2 *X* (for *n* = 1). These systems exhibit an atomically laminated structure composed of *M-X-M* (*M*_2_*X*) slabs interleaved by *A*-element atomic layers. The atomic layers are stacked along the *c*-axis. The layered, highly anisotropic crystal structure results in mechanical properties usually associated with ceramics, such as high stiffness, damage tolerance and resistance to corrosion and thermal shock^1,2^. The chemical bonding of the *M*, *A* and *X* elements is anisotropic and comprises metallic, covalent and ionic character^2^. The strong hybridization between *d* orbitals of the *M*-element and 2*p* states of the *X*-element results in directed covalent bonds along the *M-X-M* chains in basal planes^2,3^. The *M-A* bonding is generally weaker and accompanied by partial charge transfer from the *M*-element to the *A*-element, giving rise to the ionic contribution^2–4^. Metallic-like bonding between *d* states of the *M*-element occurs in the vicinity of the Fermi level (*E*_*F*_). These states dominate the density of states (DOS) at *E*_*F*_ and contribute to the electrical conductivity^2–7^. The anisotropy of the electronic structure leads to an anisotropic conductivity: higher conductivity within the *a-b* basal plane and lower conductivity along the *c*-axis. Due to partial charge transfer from the *M*-element towards the *A*-site, MAX phases are also considered as two-band conductors, where positive charges (holes) are mainly responsible for the higher conductivity in the basal plane and negative charges (electrons) mainly contribute to the lower conductivity along the *c*-axis^6^. Remarkably, the bond strength and valence electron population are strongly dependent on the choice of *A*-element. This offers the possibility of tailoring the electronic structure and, accordingly, the physical properties of MAX phase materials^2^.

**Figure 1:**
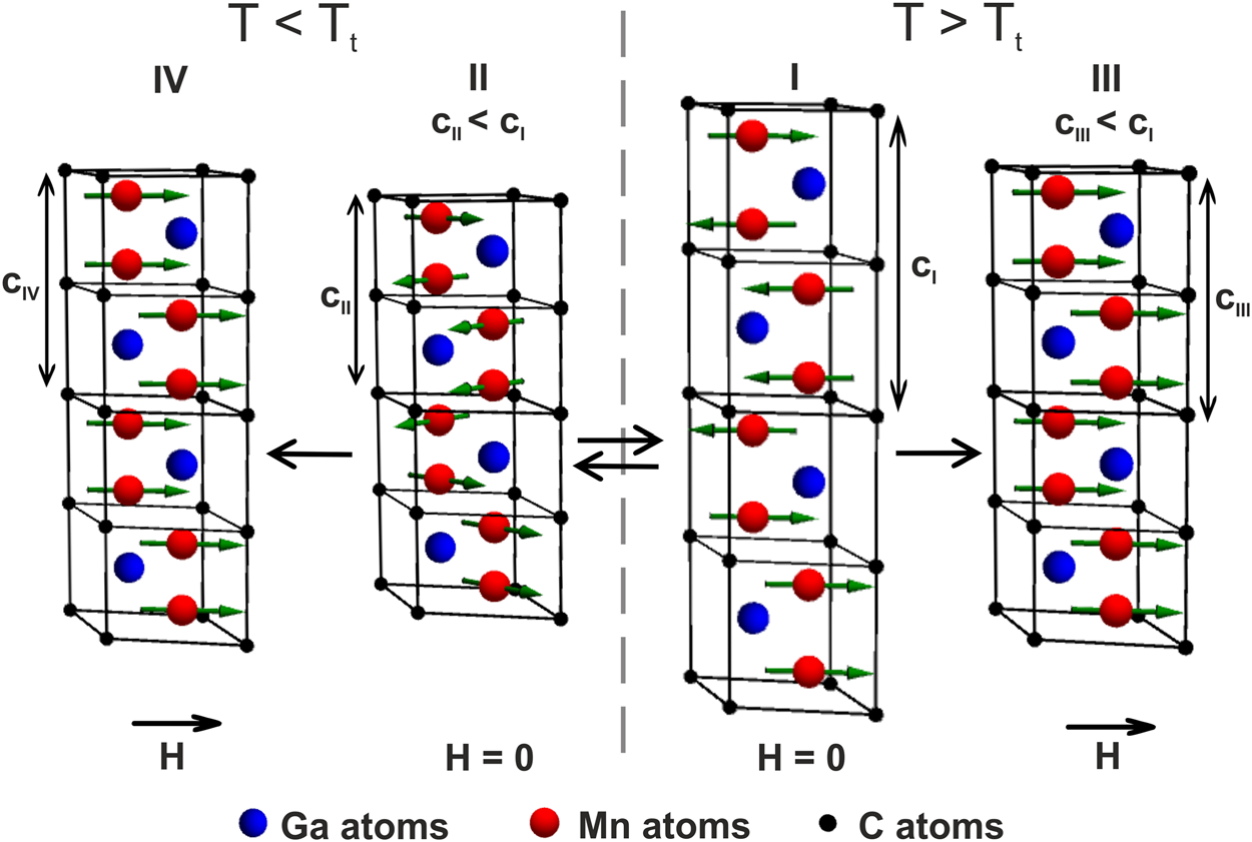
Schematic representation of magneto-structural transformations in the Mn_2_GaC film as a response of temperature and external magnetic field applied parallel to the film plane. (**I**) At *T* > *T*_*t*_ = 214 K the system has an AFM [0001]_4_^A^ magnetic spin configuration and respective spin alignment between magnetic sublattices is collinear^11–13^. (**II**) At *T* < *T*_*t*_ = 214 K the Mn_2_GaC undergoes the first order phase transition, which is characterized by *c*-axis lattice compression (c_II_ < c_I_) and magnetic spin transformation to a non-collinear AFM [0001]_4_^A^ state. (**III**) at *T* > *T*_*t*_ an external magnetic field causes the parallel spin alignment and compression of the lattice along the *c*-axis (c_III_ < c_I_) delivering large negative magnetostriction of −450 ppm. (**IV**) At *T* < *T*_*t*_ parallel spin configuration causes large positive magnetostriction of 450 ppm (c_IV_ > c_II_).

The discovery of new Mn-based magnetic MAX phases^8^ provides an additional magnetic degree of freedom to the aforementioned anisotropic structural and electronic properties. The new stable Mn_2_GaC compound has been theoretically predicted and subsequently grown as hetero-epitaxial films on MgO(111) substrates^9,10^. Theoretical studies predict a compound that shows magnetic order at room temperature and a critical order-disorder temperature that is very sensitive to the number of coordination shells used in the calculation, is approximately 660 K but with a 20% uncertainty^11,12^. The spin structure and magnetic behavior in this material is complex as seen from first-principles calculations^11–13^, magnetometry^8,9,11^ and neutron reflectometry studies^8,13^. Density functional theory (DFT) calculations suggest strong ferromagnetic coupling between Mn atoms in a Mn-C-Mn (*M-X-M*) chain^11,13^. The distance between neighboring Mn atoms in these chains is about 0.267 nm, which is below the threshold of 0.288 nm for FM favored coupling in many Mn-compounds^11^. The FM ordering in the atomic *M-X-M* trilayers has been experimentally demonstrated using ferromagnetic resonance (FMR) measurements in quaternary MAX phases (Cr_0.5_Mn_0.5_)_2_GaC^14,15^ and (Mo_0.5_Mn_0.5_)_2_GaC^16,17^. Antiferromagnetic (AFM) and FM interactions across the Ga atomic layers in Mn-Ga-Mn chains are calculated to be competitive, suggesting a strong dependence of the magnetic configuration on pressure (*c*-axis lattice parameter), temperature (*T*) and external magnetic fields (*H*)^8,11,12^. The calculated lowest energy AFM structure, which competes with the FM ordering, has the magnetic spin configuration denoted as AFM ${[0001]}_{4}^{A}$ in refs^8,11–13^, i.e. 4 consecutive *M*-layers with the same spin direction before changing sign upon crossing an *A* layer, Ga in this case. Consequently, AFM ${[0001]}_{4}^{A}$ indicates a doubling of the magnetic period as compared to the structural period along the *c*-axis as demonstrated schematically in Fig. 1(I). Neutron reflectometry (NR) confirms the long-range AFM ${[0001]}_{4}^{A}$ structure showing a magnetic periodicity of two structural unit cells^13^. The consequence of such an AFM structure is the larger interlayer distance between the AFM coupled Mn_2_C slabs across the Ga as compared to the distance between those Mn_2_C slabs, which are FM coupled^11^. Magnetometry at low temperatures shows that Mn_2_GaC is magnetically not saturated in a field of 5 T and the remanent magnetization *M*_*R*_ relative to the magnetization *M* at 5 T is small^11^. The small *M*_*R*_ and the large magnetic saturation field further support the AFM configuration as deduced from NR. However, the nonzero *M*_*R*_ and a coercive field of about 20 mT suggest the presence of a FM component in the magnetic structure. A reasonable compromise for the magnetic structure based on the observed AFM alignment by NR and the FM component from magnetometry is a canted AFM configuration^13^. A canted AFM structure means that the magnetic moments of the Mn_2_C slabs across the Ga layers are not collinear and the angle between AFM coupled moments is below 180° and is schematically represented in Fig. 1(II). In such a configuration, the projected collinear AFM components can be detected using NR and, at the same time, both magnetic sublattices project a small FM component along the measurement axis delivering the observed nonzero net magnetization^11,13^. Consequently, the Zeeman energy in the applied magnetic field favors a decreased angle between canted AFM moments and the magnetic structure evolves toward a parallel FM alignment at larger fields^11^. At *T* > 210 K Mn_2_GaC undergoes a magnetic phase transition characterized by a significant increase of the *c*-axis lattice parameter (0.1%) with increasing temperature and a decrease of *M*_*R*_^11^.

The competing exchange interactions across the Ga layers in the quasi two-dimensional Mn_2_GaC suggest a frustration of the magnetic configurations, which is mediated by the *c*-axis lattice parameter and hence the electronic structure. The influence of the magnetic order on the electronic structure in Mn_2_GaC has the potential to deliver new functionalities for spintronic, multiferroic, magnetoelectric and magnetocaloric applications by enabling the manipulation of electronic and lattice structure with a magnetic field. To obtain an experimental manifestation of the coupling between the spin, lattice and electronic structure, we performed a comprehensive suite of studies exploring the magnetic phase transitions, structural transformations, electrical transport and magnetic properties in Mn_2_GaC hetero-epitaxial films. Two magnetic phase transitions at 214(5) K and 507(5) K were identified. *T* = 507 K is the Néel temperature at which point the system changes from the AFM to the paramagnetic (PM) state. In the AFM state, the films exhibit large *c*-axis compressive (negative) magnetostriction of −450 ± 40 ppm at *T* = 280 K and 3 T. At *T* = 200 K, i.e. below the first order phase transition, Mn_2_GaC is characterized to be in a canted AFM state with a reduced *c*-axis parameter. The uniaxial magnetostriction changes sign and becomes tensile with the value of 450 ± 40 ppm at 3 T. The change of sign at the magnetic phase transition is also identified in the magnetoresistance being negative (−3%) at 300 K and positive (3.7%) at 10 K and 9 T. These findings point towards a strong coupling among magnetic, electronic and lattice structure in the layered Mn_2_GaC MAX phase. For further exploration of its functional properties for applications we discuss its magnetocaloric properties at 214 K.

## Results

### Structural analysis

**Figure 2:**
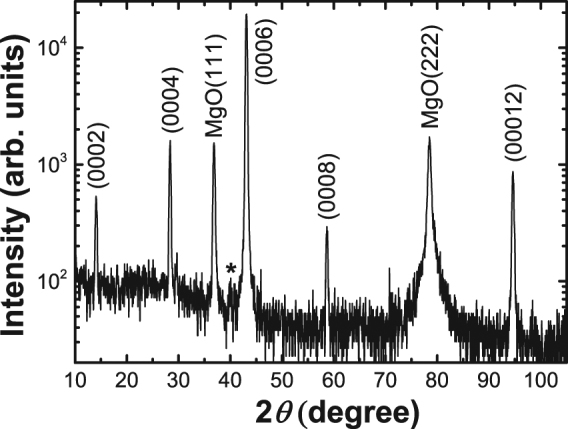
θ-2θ scan of a Mn_2_GaC thin film revealing an essentially phase pure sample, as only Mn_2_GaC and MgO substrate peaks are visible. Trace amounts of Mn_3_GaC can also be seen as a small (111) peak around ~40°, marked with asterisk.

Figure 2 shows X-ray diffraction of the Mn_2_GaC epitaxial thin film grown on MgO(111). Only reflections originating from the basal planes of Mn_2_GaC alongside the substrate are seen in the diffractogram. Trace amounts of a Mn_3_GaC antiperovskite may be present if the small (111) peak marked with an asterisk in Fig. 2 is statistically meaningful. The Mn_2_GaC lattice parameter, determined from the diffractogram, is *c* = 1.255 ± 0.001 nm. The in-plane lattice parameter *a* = 0.290 ± 0.001 nm, was determined from the $(10\bar{1}3)$ peak, acquired by tilting and rotating the sample.

**Figure 3:**
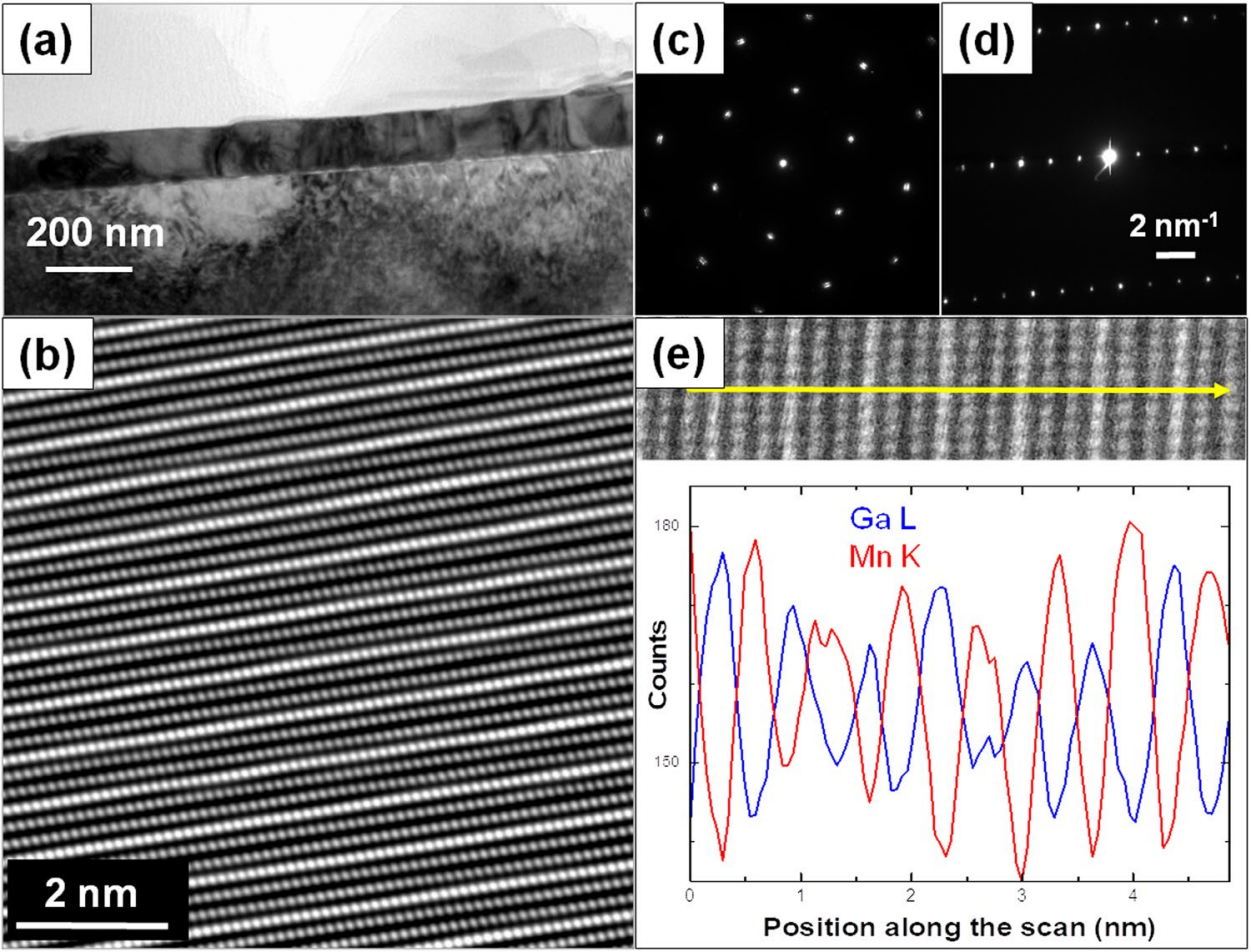
(**a**) Cross-sectional bright field TEM image of the Mn_2_GaC film on MgO(111) substrate; (**b**) HAADF/STEM atomic resolution image of the structure. The bright (grey) points correspond to Ga (Mn) atomic columns; (**c**) [0001] and (**d**) $[01\bar{1}0]$ zone axis selected area electron diffraction patterns recorded in plane-view and cross-section of the sample; (**e**) Atomic plane resolved distribution of Ga and Mn in the Mn_2_GaC obtained by the line-scan analysis using STEM/EDX along the yellow arrow as shown in the corresponding HAADF/STEM image.

Figure 3(a) shows a low magnification cross-sectional bright-field transmission electron microscopy (TEM) image of the uniform Mn_2_GaC film with a thickness of about 100 nm on a MgO(111) substrate. The cross-sectional high-angle annular dark-field scanning TEM (HAADF/STEM) atomic resolution image in Fig. 3(b) illustrates the high quality, atomically layered structure with alternating one Ga atomic layer (bright) with two Mn (darker) layers. Such a layered structure is typical for MAX phase compounds^9,14,16^. The compositional uniformity is confirmed by STEM energy-dispersive X-ray (STEM/EDX) line-scan analysis shown in Fig. 3(e), where the ordered alternating two Mn and one Ga atomic plane arrangement along the *c*-axis is also clearly identified. The high crystal quality and structure of the sample is confirmed by the selected area electron diffraction pattern for [0001] zone axis recorded in plane-view sample (Fig. 3(c)) and $[01\bar{1}0]$ zone axis for the cross-section sample (Fig. 3(d)). Indexing of electron diffraction pattern is presented in Supplementary Fig. S1. The lattice parameters determined from electron diffraction are *c* = 1.26 ± 0.01 nm and *a* = *b* = 0.294 ± 0.01 nm, which are in good agreement with the values determined from XRD measurements. No evidence of the antiperovskite phase was seen in the electron diffraction patterns.

### Magnetization

**Figure 4:**
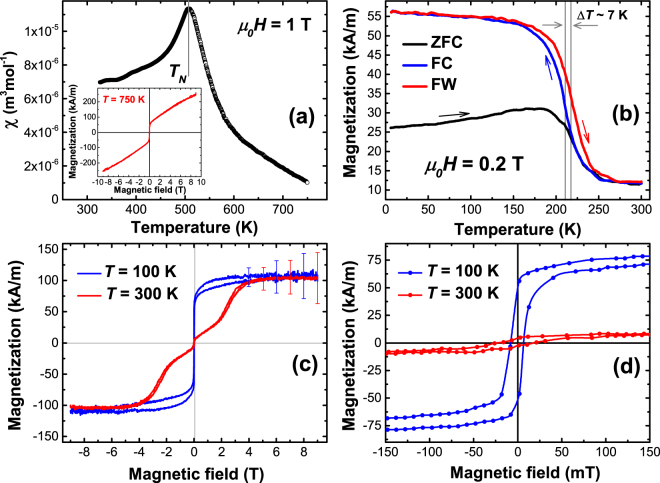
(**a**) Temperature dependence of the static magnetic susceptibility measured at 1 T field applied parallel to the Mn_2_GaC film plane. The inset shows the field dependence of the magnetization at 750 K. (**b**) Zero-field-cooled (ZFC, black), field-cooled (FC, blue) and field-warmed (FW, red) magnetization curves at magnetic field of 0.2 T applied parallel to the film surface. Arrows with corresponding color represent the sweeping direction. (**c**) Magnetization curves for Mn_2_GaC at 100 K (below the first order magnetic phase transition temperature *T*_*t*_ = 214 K, blue) and 300 K (above *T*_*t*_, red). The error bars show the uncertainty of the slope subtraction arising from the large diamagnetic contribution of the MgO(111) substrate. (**d**) The low magnetic field part of the magnetization from hysteresis loops shown in (**c**). The magnetic signals of the MgO(111) substrate were subtracted in all presented curves.

The static magnetic susceptibility *χ* = *M/H* as function of temperature in a 1 T field applied parallel to the film plane is shown in Fig. 4(a). We observe a broad, but distinct peak with a maximum at *T* = 507(5) K. This peak suggests a phase transition from the AFM state (concluded from NR measurements^13^) to a disordered state, with a Néel temperature of *T*_*N*_ ~ 507 K. The determined Néel temperature is within the range predicted theoretically^12^. The susceptibility curve above *T*_*N*_ (507 K–750 K), however, deviates significantly from the Curie-Weiss behavior suggesting that the system at these temperatures is not in a simple PM state. More reliably, this is seen from the nonlinear behavior of the *M*(*H*) curve at 750 K, shown in the inset of Fig. 4(a). The curve does not show the typical behavior for Langevin or Pauli paramagnetism. The deflection of the *M*(*H*) at a magnetic field of about 0.1 T indicates a field induced magnetic transition, a feature usually seen in itinerant electron magnetic materials with layered crystal structures^18–20^. The detailed analysis of the magnetic behavior at these temperatures remains for future studies. However, it should be noted that above 800 K the Mn_2_GaC decomposes to other phases.

The magnetization behavior at low temperatures and in a magnetic field of 0.2 T applied parallel to the film plane is shown in Fig. 4(b). Another magnetic phase transition is seen at approximately 214(5) K and there is a thermal hysteresis of *∆T* ≈ 7 K in field-cooled (FC) and field-warmed (FW) curves. The thermal hysteresis is a result of structural transformation upon the magnetic phase transition, which was identified in ref.^11^ by the 0.15% *c*-axis lattice contraction upon warming from 150 K to 300 K. The phase transition is rather broad starting from 180 K and ending at 240 K. The transition temperature *T*_*t*_ = 214 K we define by averaging inflection point temperatures in FC and FW curves. At larger magnetic fields *T*_*t*_ increases and reaches ~230 K in 1 T, while the opening of the hysteresis remains constant *∆T* ≈ 7 K. The observed characteristics suggest the phase transition to be of the first order. However, detailed studies are necessary for direct evidence.

The zero-field (ZFC) and field (FC) cooled *M*(*T*) curves (Fig. 4(b)) show noticeable splitting below 214 K. This splitting cannot be attributed to thermal fluctuations due to the absence of a characteristic hump in the FC curve and high Néel temperature of Mn_2_GaC. The thermally activated magnetic transformations are seen only in ZFC curve as a slight increase in magnetization with rising temperature from 6 K to ~190 K. The split of ZFC-FC curves in Fig. 4(b) is very similar to systems with competing FM and AFM exchange interactions such as Mn_3_Sn^20–22^. We note that the temperature dependent magnetization curves in Fig. 4(b) do not show any visible magnetic response from the Mn_3_GaC antiperovskite, which has an AFM – FM first order phase transition at 160 K and FM-PM second order transition at 250 K^23,24^.

In order to identify the difference in magnetic states below and above *T*_*t*_, hysteresis loops were measured at 100 K and 300 K and are presented in Fig. 4(c), with an enlarged view at low fields shown in Fig. 4(d). Both curves exhibit a plateau at magnetic fields above 6 T with a magnetic moment of 0.33 ± 0.15 *μ*_*B*_ per Mn atom. This magnetization is significantly smaller than predicted theoretically^11^ and four times smaller than in the (Mo_0.5_Mn_0.5_)_2_GaC quaternary MAX phase^16,17^. This suggests that at both temperatures the Mn magnetic moments are not parallel and much larger fields are necessary to transform the non-collinear AFM configuration into a collinear FM. This is also supported by the results in the inset of Fig. 4(a). At 750 K and 9 T the magnetization is 0.75 *μ*_*B*_ per Mn atom, which is significantly larger than the magnetization at either *T* = 100 K or *T* = 300 K. Additional evidence for non-FM alignment at temperatures below 214 K is the difference between magnetization values at 9 T measured after ZFC and FC as shown in Supplementary Fig. S2. The irreversibility between descending and ascending branches of the *M*(*H*) curves in Fig. 4(c) is the result of structural changes upon application of the external field. The shape and irreversibility of *M*(*H*) curves are discussed below. In both AFM states the system exhibits a net spin magnetization as seen in the enlarged view in Fig. 4(d). The origin of this weak ferromagnetic moment is likely the canting of Mn moments in the different AFM sublattices towards the field direction^8,11–13^, an effect observed in the structurally similar Mn_3_Sn which is also a non-collinear antiferromagnet^20–22^. The different shape and remanent magnetization of the hysteresis loops at 100 K and 300 K in Fig. 4(c),(d) suggest that *T*_*t*_ = 214 K is the spin reorientation temperature, at which the magnetic structure changes from a collinear AFM state at *T* > *T*_*t*_ to a non-collinear AFM configuration at *T* < *T*_*t*_. This transition from state (I) to state (II), as indicated in Fig. 1, is accompanied by a *c*-axis lattice compression^11^. The magnetic spin transformation is also seen from NR measurements as a splitting and broadening of the “AFM peak” at 290 K as compared to the peak at 150 K^13^. Besides AFM ${[0001]}_{4}^{A}$, an additional possible AFM structure with slightly different propagation vector has been suggested^13^, though the exact spin configuration of Mn_2_GaC films is an open topic for future studies using NR with fine field and temperature resolution.

### Magnetoresistance

**Figure 5:**
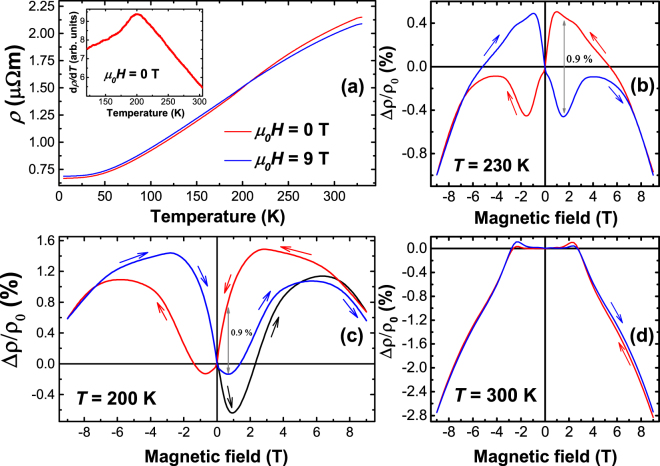
(**a**) Temperature dependence of electrical resistivity measured in zero field (red) and 9 T field (blue) applied parallel to the current direction in the Mn_2_GaC film plane. The inset shows the first derivative of the electrical resistivity in zero field. Panels (**b**–**d**) show the magnetoresistance measured at 230 K, 200 K and 300 K relative to the zero field value. Arrows represent the sweeping direction.

The temperature dependence of the electrical resistivity *ρ* of a 100 nm thick Mn_2_GaC film is shown in Fig. 5(a). The resistivity decreases with decreasing temperature showing overall metallic behavior. At room temperature the electrical resistivity of *ρ* = 2.0 μΩm is larger as compared to (Mo_0.5_Mn_0.5_)_2_GaC films which show *ρ* = 1.5 μΩm, even accounting for the relatively large systematic errors in the measurements^17^. At low temperatures, however, the resistivity of the Mn_2_GaC film is noticeably smaller and, accordingly, the residual resistivity ratio (RRR = ρ(300 K)/ρ(10 K) = 3.1) is larger than for the quaternary (Mo_0.5_Mn_0.5_)_2_GaC (RRR = 1.3)^17^. These results are in line with Lin *et al*.^25^, suggesting that the influence of structural imperfections on electrical properties becomes stronger when alloying *M*-elements in MAX phase quaternaries. At high temperatures (100 K–330 K) the resistivity does not show the simple linear temperature dependence as it shows in the non-magnetic MAX phase ternaries due to additional phonon contributions^25,26^. The origin of non-monotonic temperature dependence can be understood by inspecting the derivative d*ρ/*d*T*, shown in the inset of Fig. 5(a). d*ρ/*d*T* shows a broad, but clear maximum near the first order phase transition temperature *T*_*t*_ (214 K), indicating the strong influence of the magneto-structural transformations on the transport properties. This is also seen when comparing *ρ*(*T*) curves (Fig. 5(a)) measured in zero field (ρ_0_) and 9 T (ρ_9T_) applied parallel to the current direction and in the film plane. The magnetoresistance (MR) at 9 T, Δ*ρ*_9T/ρ__0_ = (*ρ*_9T_ − ρ_0_)/ρ_0_, which is negative (Δ*ρ*_9T_/ρ_0_ = −3%) at 300 K changes sign at *T*_*t*_ and becomes positive reaching the value of Δ*ρ*_9T_/ρ_0_ = 3.7% at 10 K. The MR at selected temperatures is shown in Fig. 5(b–d). At temperatures below *T*_*t*_ (Fig. 5(c)) the MR shows a complex, non-monotonous field dependence with an irreversibility point at about 7 T. In contrast to (Mo_0.5_Mn_0.5_)_2_GaC^17^ the MR in Mn_2_GaC is hysteretic, showing a high asymmetry with respect to the inversion of the field direction. This asymmetry is a result of magneto-structural transformations, which lead to different electronic structures. For example, at +0.7 T (Fig. 5(c)) the asymmetry of MR between the “ +9 T curve” (red) and the “−9 T branch” (blue) reaches a value of 0.9%. More interestingly, at small positive fields the “ +9 T curve” shows positive MR, whereas for the “−9 T curve” the MR is negative. This allows the read out of the magneto-structural states even at fields below 0.7 T by detecting the sign of the MR.

Similar to the magnetization, the MR is also influenced by the AFM frozen states after the ZFC from 330 K down to temperatures below *T*_*t*_. This is seen as a significant difference of the MR measured right after cooldown (black curve in Fig. 5(c)) in comparison with +9 T (red) or −9 T (blue) curves. The mismatch between black and blue curves in Fig. 5(c) indicates that at 200 K the Mn_2_GaC film has different electronic structure (charge carriers concentration and density of states at the Fermi level) as compared to the structure stabilized after application of a 9 T magnetic field. This difference is the consequence of irreversibility of the lattice and magnetic structures. The irreversibility is also identified in magnetization curves, which show different remanence before and after application of 9 T fields. However, the shape of the black and blue curves in Fig. 5(c) is similar indicating qualitatively similar magneto-structural transformations as a response on magnetic field.

At temperatures above *T*_*t*_ the complex non-monotonous behavior of the MR remains with modified shape and asymmetry values at different fields as seen in Fig. 5(b). At 9 T, the MR in Fig. 5(b) is negative, which is in agreement with the sign inversion of MR across *T*_*t*_ in Fig. 5(a). At higher temperatures the irreversibility field decreases, the asymmetry is reduced and the MR evolves mainly towards negative values (Fig. 5(d)). Qualitative understanding of the MR as well as the magnetization behavior as a function of applied magnetic field can be gained by plotting them together with the field dependent magnetostriction, which is discussed in the next section.

### Magnetostriction

**Figure 6:**
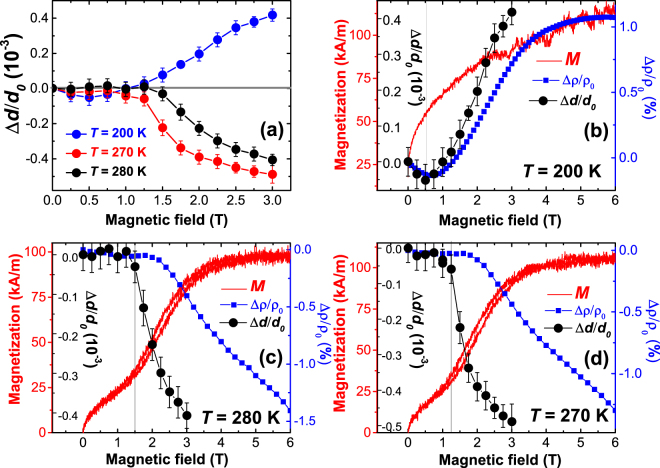
(**a**) Field dependence of magnetostriction measured at 200 K (blue), 270 K (red) and 280 K (black) in a field applied parallel to the Mn_2_GaC film plane. Panels (b,c,d) combine plots of MS (black circles and left inner scale bar), magnetization (red line and left outer scale bar) and MR (blue squares and right scale bar) measured at (**b**) 200 K (below *T*_*t*_), (**c**,**d**) 280 K and 270 K (above *T*_*t*_).

The influence of the magnetic field on the lattice parameter was studied using high resolution double-axis XRD, where the Mn_2_GaC (0006) Bragg reflections (see Supplementary Fig. S3) were recorded under different magnetic fields applied parallel to the film plane. In Fig. 6(a) the relative change of the inter-plane distance ∆*d* with respect to the d-spacing at zero field *d*_*0*_ is plotted. Thus, Fig. 6(a) shows the temperature dependence of the *c*-axis magnetostriction which is along the film normal. One sees that, similar to the MR, the magnetostriction (MS) at temperatures above *T*_*t*_ = 214 K is negative, indicating a lattice compression with increasing magnetic field. The MS changes sign to positive values at *T* < *T*_*t*_, showing a tensile strain in response to the applied magnetic field. Besides the unusual sign inversion of MS at temperatures above and below *T*_*t*_, the absolute value of (0.45 ± 0.04) × 10^−3^ (450 ± 40 ppm) at 3 T is large in both cases (Fig. 6(a)). Such a high value of MS indicates that the FM alignment of the magnetic moments within the Mn_2_GaC compound leads to significant decrease (increase) of the *c*-axis lattice constant across the *T*_*t*_ transition temperature (as schematically sketched in Fig. 1). We also note that the MS shows a noticeable temperature dependence as indicated by the vertical gap between the 270 K and 280 K curves in Fig. 6(a).

The observed large MS is the consequence of the field-induced transitions from AFM or non-collinear AFM spin states towards FM configuration. To gain a comprehensive view on the magneto-structural transformations in the system we refer to Fig. 1. The largest *c*-axis lattice compression by 0.12% arises across the phase transition temperature even at zero magnetic fields: from (I) to (II) in Fig. 1. The FM alignment of magnetic moments leads to compression by 0.045% at *T* > *T*_*t*_: from (I) to (III) and stretching by 0.045% at *T* < *T*_*t*_: from (II) to (IV). The lattice compression below *T*_*t*_ (from (I) to (II) in Fig. 1) is 2.6 times larger than both, negative and positive magnetostrictions. The sign inversion of the MS at *T*_*t*_ suggests that the sign of exchange coupling between Mn moments across the Ga atomic layers has oscillatory-like behavior as function of the *c*-axis lattice parameter (see Supplementary Fig. S5). It should be noted that the in-plane lattice parameter does not change significantly upon the *c*-axis lattice modifications, thus the magneto-structural transformations near the first order phase transition is accompanied by a change of the unit cell volume^11^.

For direct comparison it is helpful to plot the field dependence of the magnetization, magnetoresistance and magnetostriction in one panel as is shown in Fig. 6(b–d). First we discuss the case *T* > *T*_*t*_, shown in Fig. 6(c,d). At magnetic fields up to 1.5 T at 280 K (Fig. 6(c)) or 1.3 T at 270 K (Fig. 6(d)) the MS does not show a visible field dependence indicating that the *c*-axis lattice constant does not change. In this region the magnetization shows an almost linear response to the magnetic field indicating that the magnetic field leads to a canting of the magnetic moments and provides the nonzero net magnetization without any noticeable change in the crystal lattice. At magnetic fields above 1.5 T the magneto-structural transformation sets in as seen from the compression of the atomic planes (increase in negative MS) and the magnetization shows a rapid increase up to a saturation at ~6 T. The rapid increase in magnetization is accompanied by a small hysteresis seen in Fig. 6(c) and (d), which is the result of structural transformations.

At *T* = 200 K (Fig. 6(b)), i.e. *T* < *T*_*t*_ and MS is positive, the magnetization has the opposite behavior. First, it shows a rapid increase and then it slows down at magnetic fields where further magnetic spin alignment towards FM state causes lattice tension (increase of MS). This indicates that at low magnetic fields (below 1 T in Fig. 6(b)) the Zeeman energy helps to align the spin structure to be parallel to the field direction microscopically as well as macroscopically in case of the presence of magnetic domains. At a certain field, the ongoing FM spin alignment requires a larger distance between Mn_2_C slabs across the Ga atomic layers resulting in an expansion of the lattice. The obtained magneto-structural transformations are in line with the DFT calculations^10,15,16^, which predict a strong influence of the magnetic configuration on the *c*-axis lattice parameter in this system with competing AFM and FM interactions across the Ga atomic layers.

Electrical conductivity also shows a noticeable response to the magneto-structural transformations. Figure 6(c,d) show that the compressive *c*-axis strain (negative MS) leads to a decrease of the electrical resistivity delivering −1.4% of MR at *T* > *T*_*t*_ and 6 T field. This indicates that the decrease of the *c*-axis lattice parameter leads to a higher concentration of the charge carriers at *E*_*F*_ and, accordingly the decrease of resistivity. Although the magnetization and the MS show good correlation as a function of field, MR in Fig. 6(c,d) starts decreasing at larger fields. This delay in response of the MR is a direct result of the formation of lattice imperfections when structural deformations begin. The decrease of the carrier mobility due to enhanced lattice imperfections compensates the contribution from the increased carrier concentration due to lattice compression ensuring that the MR remains almost constant up to 2 T. At the point when the MS approaches its saturation, the MR begins to decrease as the increased carrier concentration dominates. The positive MS, which arises due to the tensile strain for temperatures below *T*_*t*_ (Fig. 6(b)) results in an increase of the interlayer distance, and the charge carrier concentration decreases, as seen in Fig. 6(b), where the MR reaches +1% at 6 T. This simplified picture of changing the carrier concentration at the Fermi level qualitatively explains the sign change of MR whilst crossing the first order phase transition temperature *T*_*t*_ = 214 K. However, spin dependent scattering of charge carriers must also be involved in Mn_2_GaC, similar to what has been found for (Mo_0.5_Mn_0.5_)_2_GaC thin films^17^. In Fig. 6(b) the rapid increase of the magnetization below 1 T with negligibly small MS is accompanied by negative MR of about −0.1%, which is still smaller than the positive MR caused by *c*-axis lattice tension at larger magnetic fields.

### Magnetocalorics

**Figure 7:**
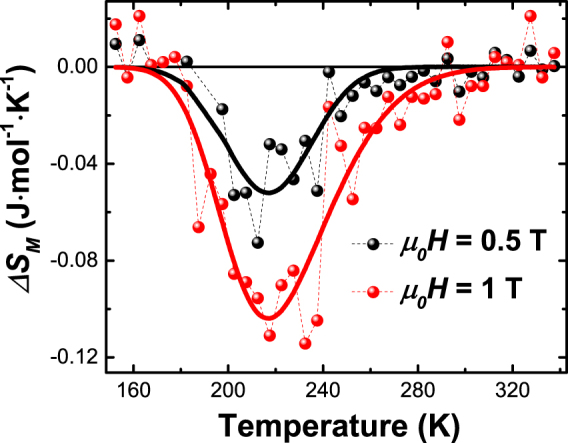
Magnetic entropy change ∆*S*_*M*_ of Mn_2_GaC film for the field changes of 0–0.5 T (black) and 0–1 T (red) calculated from magnetization isotherms. Solid lines are served as a guide to the eye.

The first order magnetic phase transition at *T*_*t*_ = 214 K (Fig. 4(b)), which accompanies the change of the electronic structure is particularly interesting for magnetocaloric applications^27^. We measured standard magnetization isotherms (shown in Supplementary Fig. S6) in order to estimate the magnetic contribution to the entropy change ∆*S*_*M*_ at the phase transition^23,27^. The temperature dependence of the magnetic entropy change calculated for two different magnetic fields is shown in Fig. 7. The sign of the ∆*S*_*M*_ is negative, since the high magnetization state is found at low-temperatures and an external magnetic field decreases the entropy as is the case in many materials with conventional magnetocaloric effects (MCE)^27^. The MCE is largest near the transition temperature. The maximum of |∆*S*_*M*_| at 1 T fields is about 0.1 J·mol^−1^·K^−1^, which is one to two orders of magnitude smaller than in materials with large MCE^23,24,27^. The reason for the small magnetic entropy change in the Mn_2_GaC film is the broad phase transition, which starts around 180 K and ends at about 240 K (Fig. 4(b)).

## Discussion

Long-range magnetic order is stabilized by including Mn as the sole *M*-element in the Mn_2_GaC MAX phase. The exchange correlations of magnetic atoms are involved in the Mn-Ga-Mn (3*d*–4*p*) bonding, giving Mn-Ga-Mn magnetic super-exchange, where the sign of the exchange integral balances the strength of the 3*d*–4*p* bonding with the *c*-axis lattice parameter^6,11^. This interplay has a strong influence on the electronic structure, coupling the spin, lattice and electrical degrees of freedom. Previously reported first principles calculations reveal that AFM and FM spin alignments across Ga atomic layers in the Mn_2_GaC compound are close to degenerate^11^. This leads to the following magnetic properties in Mn_2_GaC:

i) Non-collinear AFM states with a spin-reorientation transition and *c*-axis compression below *T*_*t*_ = 214 K.
ii) Large *c*-axis compressive/tensile magnetostriction (MS) with sign inversion across the phase transition (*T*_*t*_ = 214 K).
iii) Highly asymmetric magnetoresistance (MR) up to 3% with sign inversion at *T*_*t*_.

These magnetic properties open a pathway to explore new functionalities for practical applications. For example heating up the system to temperatures above *T*_*t*_ allows significant *c*-axis uniaxial tension by almost 0.12%. In addition to that, the system shows 450 ppm MS, whose sign can be adjusted by temperature. The electrical conductivity is also sensitive to the magneto-structural transformations, so that the sign of the MR changes near *T*_*t*_. Practically it is always challenging to determine MS, especially in thin magnetic films. The lattice strain state in Mn_2_GaC can be inferred using simpler MR measurements, a negative MR arises from lattice compression whilst a positive MR from tensile strain. The magnitude of the MR allows for the quantitative estimation of the compressed/stretched lattice. For example −450 ppm compression at 3 T field and 280 K temperature leads to a MR of −0.4% (Fig. 6(c)). The tensile strain of 450 ppm at 200 K provides a value of +0.7% MR. In turn, this means that mechanical stress along the *c*-axis, e.g. caused by external pressure, can be detected by magnetoresistance measurements. The magneto-structural transformations, which also involve modifications of the electronic structure, appear very promising for magnetic refrigeration, especially if the width of the phase transition can be narrowed. The magnetic contribution to the entropy change at the phase transition is small in Mn_2_GaC, however the significant change of the electronic structure motivates further verification of magnetocaloric effects in this system. For this, however, bulk crystals or powder samples are needed.

Among the yet discovered MAX phases, Mn_2_GaC has the largest reported magnetic ordering temperature of *T*_*N*_ ~ 507 K. This temperature is close to that predicted theoretically^12^. Due to the anisotropic structural and electrical transport properties in MAX phases it would be interesting for further studies to explore the anisotropic magnetic behavior and possible anisotropic response of the lattice and electrical conductivity to magnetic fields applied parallel and perpendicular to the *c*-axis. In addition, the studied properties can be tailored by replacing the *A*-element. Besides the search for new stable solutions of magnetic MAX phases, the *A*-element can be intercalated by substitutional solid-state reactions as was shown recently^28^. Substitution of Au for Ga in (Cr_0.5_Mn_0.5_)_2_GaC, for example, leads to an enhancement of coercive field and reduction of the ordering temperature^29^. Another perspective on mastering the magnetic properties in M_n+1_AX_n_ phases is the stabilization of 312 magnetic structures (*n* = 2). For example, the recently reported quaternary (V,Mn)_3_GaC_2_ compound shows a Curie temperature above 400 K^30^.

To conclude, the Néel temperature in the Mn_2_GaC is *T*_*N*_ ~ 507 K, at which the system changes from a collinear AFM state to the paramagnetic state. At *T*_*t*_ = 214 K the material undergoes a first order phase transition from AFM above *T*_*t*_ to a non-collinear AFM spin state. Both states show large uniaxial *c*-axis magnetostriction of 450 ppm with sign inversion at the first order phase transition. The observed magnetostriction is the consequence of the field-induced magneto-structural transformation towards FM configuration. The sign change of magnetostriction coefficient across the phase transition is a fundamentally new property, which is a consequence of the layered structure and competing antiferromagnetic and ferromagnetic exchange interactions between magnetic atomic layers in the Mn_2_GaC. The sign inversion is also found in magnetoresistance, suggesting a strong coupling among the spin, lattice and electrical transport properties.

## Methods

Mn_2_GaC thin films were deposited using magnetron sputtering from three elemental sources placed confocally: manganese (99.95% purity), carbon (99.99% purity) and gallium (99.9995% purity). For gallium, due to the low melting point (30 °C), a crucible was used to avoid gallium leakage. Details of the preparation of the Ga target, the synthesis procedure, and materials optimization can be found in references^9,10,31^, respectively. The base pressure in the ultra-high vacuum (UHV) chamber was <5 · 10^−9^ Torr and an Ar pressure of 4.5 mTorr was used during deposition. Films were deposited on 10 × 10 × 0.5 mm^3^ MgO(111) substrates, that were ultrasonically cleaned in acetone, ethanol and isopropanol for 10 minutes each prior to loading into the growth chamber. The temperature of sample was kept constant at the deposition temperature of 550 °C for 60 minutes prior to deposition.

Structural and composition characterization was performed using a probe-side Cs-corrected JEOL JEM 2200FS operated at 200 kV acceleration voltage. Overview images were taken in a conventional bright-field TEM mode while the high-angle annular dark-field STEM mode was used for the high-resolution micrographs. EDX elemental line-scan was carried out in the scanning mode utilizing an Oxford X-max detector. A cross-sectional (S)TEM sample were prepared by conventional mechanical polishing followed by low-angle Ar ion milling. Plan-view (S)TEM samples were prepared by mounting a piece of sample on mechanically polished Ti half grid followed by focused ion beam (FIB) milling using a Carl Zeiss CrossBeam 1540 EsB system.

Initial structural information was obtained by X-ray diffraction (XRD) using a Panalytical Empyrean MRD system equipped with a Cu Kα source. The tube was in line focus with a hybrid mirror on the incident side and a 0.27° collimator on the diffracted side. Measurements of the magnetostriction were performed on BM28, XMaS, at the European Synchrotron Radiation Facility, Grenoble^32^. The diffractometer was configured in a high resolution double axis configuration using narrow slits (2.0 × 0.2 mm). Samples were mounted in a cryostat and remotely positioned into the beam centre for each temperature. Fields of up to ±3 T were applied in the film plane using a warm-bore superconducting magnet mounted on the diffractometer.

Electric transport and magnetic properties were measured in a Quantum Design Physical Property Measurement System (PPMS) at temperatures ranging from 2.5 to 900 K. Transport properties were measured using a standard four-probe method exploiting a custom made sample holder. 4 gold-coated spring pin contacts were used to drive currents up to 10 mA through the sample whilst measuring the voltage drop across the inner pins. For transport measurements the sample was cut into 4 × 10 mm^2^ sized pieces. Magnetoresistance measurements were performed in magnetic field applied parallel to the current direction in the film plane. Vibrating sample magnetometry (VSM) was performed on 3 × 3 mm^2^ sized samples fixed on two different PPMS sample holders for low (2.5 K–300 K) and high (300 K–900 K) temperature measurements. Variable magnetic fields *µ*_*0*_*H* up to ±9 T were used for magnetic measurements. The magnetic signals of the MgO(111) substrate were subtracted by measuring a reference substrate in identical conditions. Prior to subtraction the magnetic responses of the sample and the reference MgO substrate were normalized to the mass of corresponding pieces.

## Electronic supplementary material

Supplementary Information 
