# Supplementary material for: Large uniaxial magnetostriction with sign inversion at the first order phase transition in the nanolaminated Mn2GaC MAX phase
Source: Sci Rep. 2018 Feb 8;8:2637. doi: 10.1038/s41598-018-20903-2 (PMC5805691; doi:10.1038/s41598-018-20903-2)
Supplement: Supplementary file 1 — Supplementary Information [file 41598_2018_20903_MOESM1_ESM.pdf]

Supplementary Information

Large uniaxial magnetostriction with sign inversion at the first order phase transition in the nanolaminated Mn_2GaC MAX phase

Iu. P. Novoselova[1], A. Petruhins[2], U. Wiedwald[1,3], A. S. Ingason[2,4], T. Hase[5], F. Magnus[6,7], V. Kapaklis[7], J. Palisaitis[2], M. Spasova[1], M. Farle[1,8], J. Rosen[2], and R. Salikhov[1,9]

[1]Faculty of Physics and Center for Nanointegration (CENIDE), University of Duisburg-Essen, 47057 Duisburg, Germany.

[2]Thin Film Physics, Department of Physics, Chemistry and Biology (IFM), Linköping University, SE-581 83 Linköping, Sweden.

[3]National University of Science and Technology «MISIS», 119049 Moscow, Russian Federation.

[4]Grein Research ehf. Dunhaga 5, Reykjavik, Iceland.

[5]Department of Physics, University of Warwick, Coventry, CV4 7AL, UK.

[6]Science Institute, University of Iceland, Dunhaga 3, IS-107 Reykjavik, Iceland.

[7]Division of Materials Physics, Department of Physics and Astronomy, Uppsala University, Box 516, SE-75121 Uppsala, Sweden.

[8]Center for Functionalized Magnetic Materials (FunMagMa), Immanuel Kant Baltic Federal University, Kaliningrad, Russian Federation.

[9]Zavoisky Physical-Technical Institute, Russian Academy of Sciences, 420029 Kazan, Russian Federation.

Electron diffraction (ED) patterns of the Mn_2GaC sample taken with the electron beam incidences along the $[0001]$ and $[01\bar{1}0]$ zone axes are shown in Fig. S1 (a) and (b), respectively. ED reflections are very distinct and sharp manifesting high crystal quality of the sample. All diffraction spots are indexed in a hexagonal $P6_3/mmc$ space group revealing lattice parameters $a = 0.294 \pm 0.01$ nm and $c = 1.26 \pm 0.01$ nm.

Fig. S2 shows that the magnetization of Mn_2GaC is strongly influenced by the conditions of cooling. Cooling down the sample below the first order phase transition temperature $T_t = 214$ K in zero fields (ZFC) leads to magnetization of about 100 kA/m. The 9 T field-cooled curves (FC), where the system is cooled down in a compressed lattice state, exhibit a magnetization which is almost twice as large with a magnetic moment of about 0.6 μ_B per Mn atom. This difference in magnetizations is the consequence of system not reaching saturation, even in a 9 T field. This suggests that the system as a whole is not truly ferromagnetic, and much larger magnetic fields are required for complete magnetic saturation.

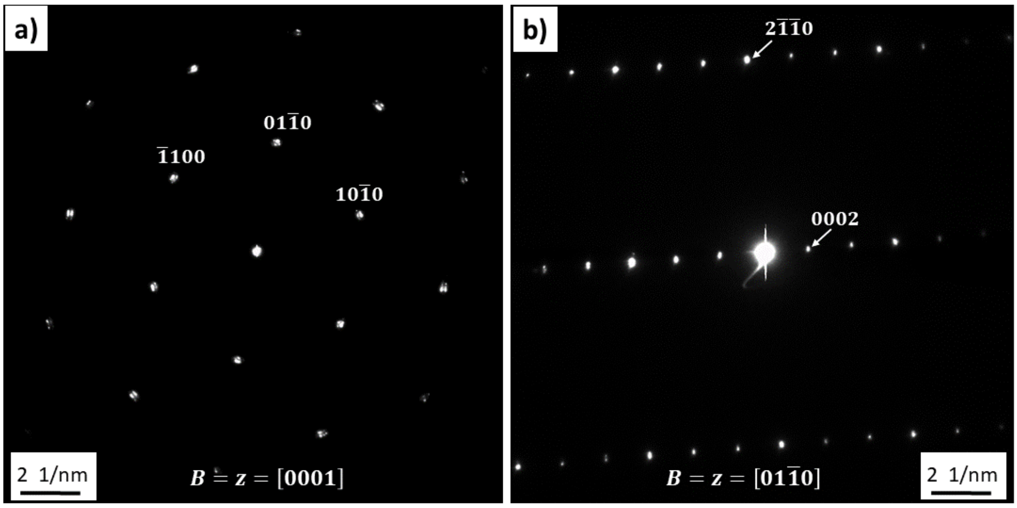

Figure S1. Selected Area Electron Diffraction (SAED) patterns taken along [0001] (a) and $[01\bar{1}0]$ (b) zone axis.

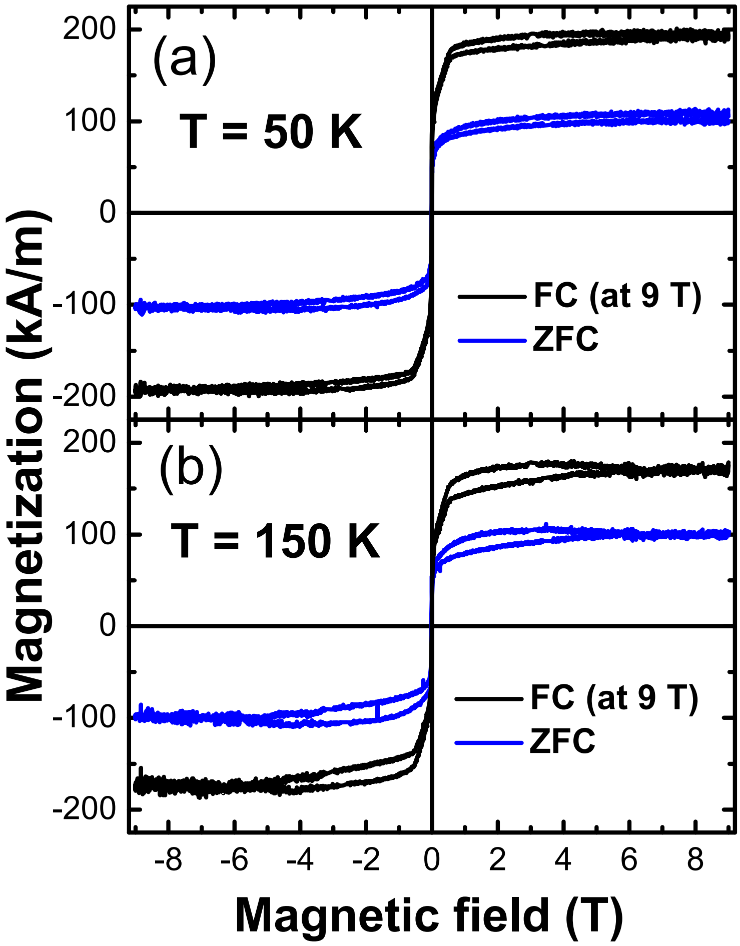

Figure S2. Magnetization curves for Mn_2GaC measured at (a) 50 K and (b) 150 K in two different regimes: after cooling down the sample to the corresponding temperature from 330 K in zero (ZFC, blue curves) and in 9 T (FC, black curves) field applied parallel to the film surface.

Fig. S3 presents the Mn_2GaC (0006) Bragg reflections as a function of magnetic field applied parallel to the film surface at 200 K (a) and 270 K (b). At 200 K the diffraction peaks shifts towards smaller scattering vectors $Q = |\mathbf{Q}|$ as the field increases showing a compression of the lattice parameter. For the 270 K data, the opposite is the case with the peak shifting to higher scattering vectors on application of the field. The magnetostriction was determined from the relative shift in peak position, and hence lattice parameter relative to zero applied field. In a slit defined geometry, small shifts in peak position can rise from sample movements which introduce height errors. To ensure the shifts in the Mn_2GaC peaks arise from magnetostriction the Bragg peak position of the MgO(111) substrate was also measured. As is evident in Fig. S4 the normalized position and hence lattice parameter of MgO is not influenced by the applied field and the calculated d-spacing at different magnetic fields remains constant as expected. The error in the peak positions (1×10^{-4}) is significantly smaller than the peak shifts seen in Fig. S3 (3×10^{-3}) showing that the MS can be determined accurately and precisely.

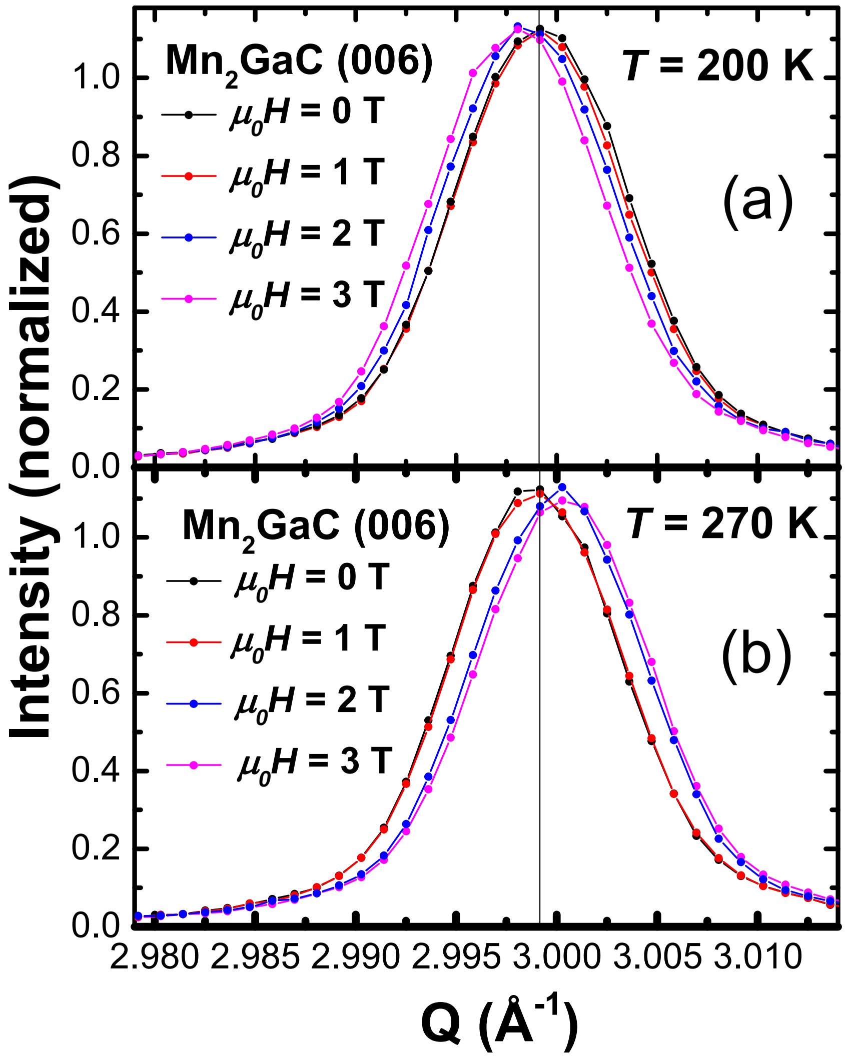

Figure S3. Comparison of the x-ray diffraction Mn_2GaC (006) peak position at different magnetic fields applied parallel to the Mn_2GaC film plane: (a) below (200 K) and (b) above (270 K) the first order phase transition temperature.

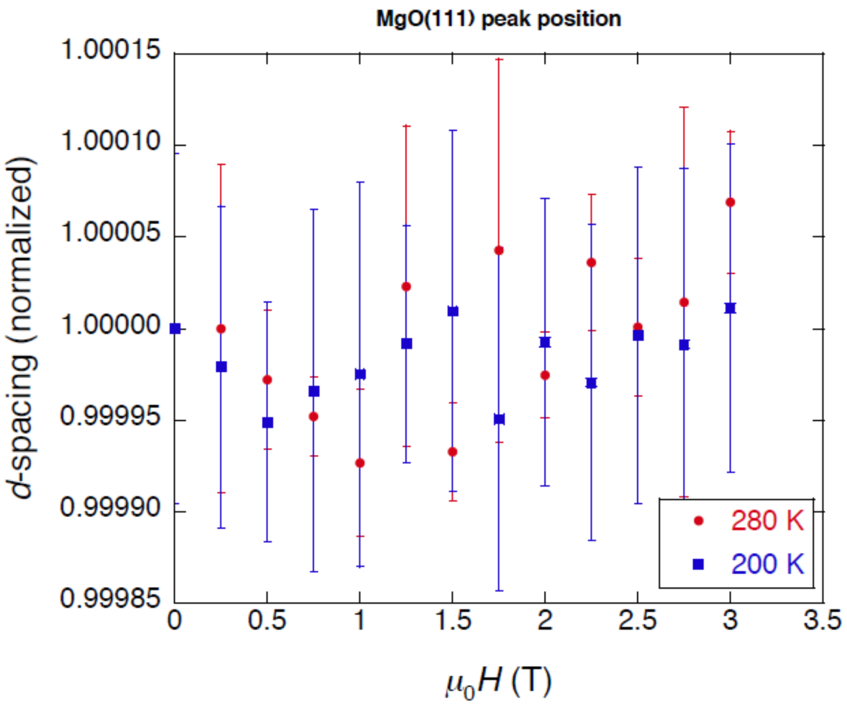

Figure S4. Magnetic field dependence of the atomic inter-plane distance (*d*-spacing) in the MgO(111) substrate calculated from MgO (111) diffraction peak position at 280 K (red circles) and 200 K (black squares).

In order to gain better knowledge of the sign change of magnetostriction across the first order phase transition, we plot in Fig. S5 the *c*-axis lattice parameter at different temperatures and fields normalized to a (reference) lattice constant at 270 K and zero magnetic field, where system is in an AFM state (denoted as AFM1 in Fig. S5). As seen in the Fig. S5, application of a 3 T magnetic field leads to a lattice compression by 0.045% at 270 K. The transition from the AFM1 state at 270 K to a non-collinear AFM at 200 K causes 0.12% compression. Higher magnetic fields at 200 K, which favor FM alignment, reduces the lattice compression down to 0.074% accounted from the reference at 270 K. This behavior suggests that the FM alignment of magnetic spins prefers intermediate *c*-axis lattice compression between the AFM1 and AFM2 states, for example near -0.06% (Fig. S5). Note that at 3 T the sample is not truly FM at both temperatures. It is straightforward to assume that the non-collinear AFM state at 200 K is an intermediate state between the FM state at 0.06% lattice compression and possible hypothetical second AFM (AFM2) state at compression larger than 0.12% with respect to the reference. Considering this assumption is valid, one can conclude that the sign of exchange coupling between Mn across the Ga atomic layers has oscillatory behavior as a function of the *c*-axis spacing. A sign change of the exchange coupling as a function of the *c*-axis lattice parameter can be expected for a layered magnetic system with competing FM and AFM interactions.

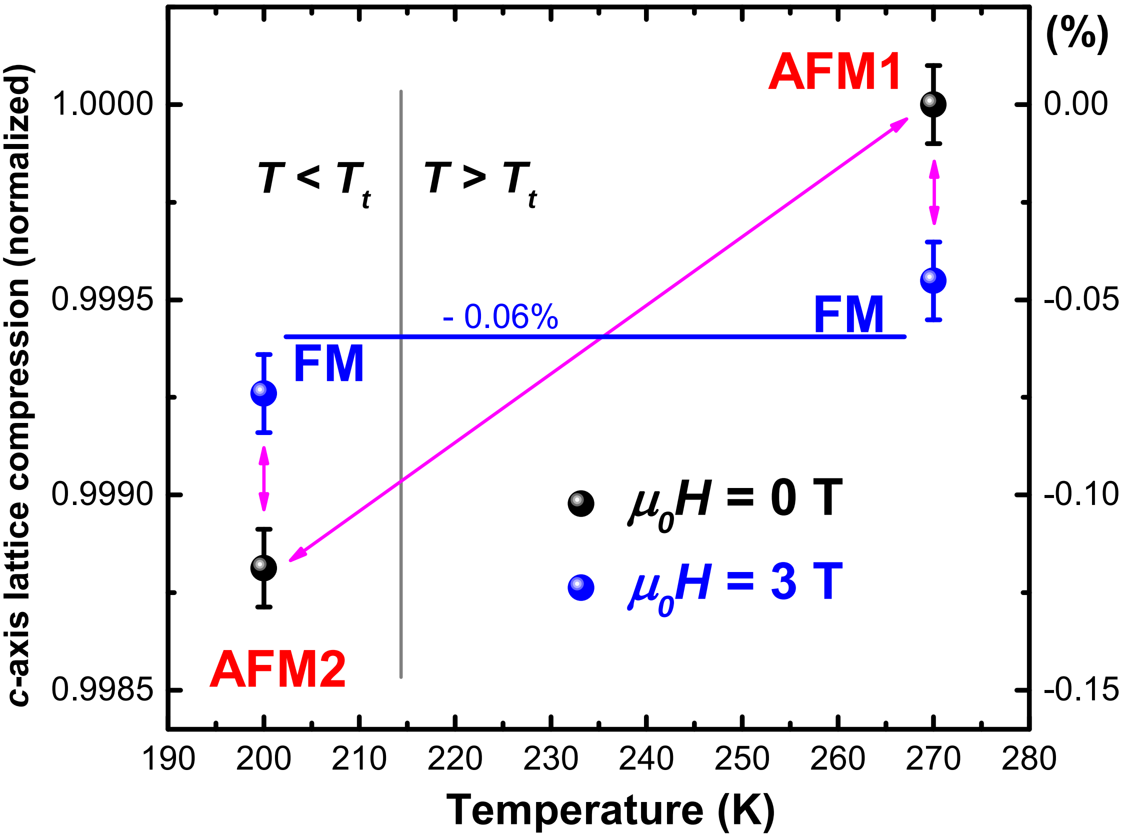

Figure S5. Relative *c*-axis lattice compression accounted with respect to the lattice parameter at 270 K and zero magnetic field. Black and blue circles represent states at zero and 3 T fields, respectively. The vertical line indicates the first order phase transition temperature (T_t = 214 K at 0 T), arrows mark directions of lattice variation as a response to the temperature or magnetic field change. Abbreviations AFM1, FM and AFM2 are used for schematic representation of expected spin structures at corresponding *c*-axis lattice strain.

Figure S6 presents magnetization curves as a function of magnetic field at different temperatures across the first order magnetic phase transition T_t. The temperature dependencies of magnetic entropy change ΔS_M in Fig. 7 were calculated using the Maxwell relation

$$\Delta S_M = \int_0^H \left(\frac{dM}{dT}\right)_H dH,$$

which can be approximated as

$$\Delta S_M\left(\frac{T_1+T_2}{2}\right) = \frac{1}{T_2-T_1}\left[\int_0^H M(T_2,H)dH - M(T_1,H)dH\right].$$

T_1 and T_2 are temperatures of two neighboring isotherms, which were measured with the step $T_2 - T_1$ = 5 K. The integration was performed for the field ranges of 0 – 0.5 T and 0 – 1 T.

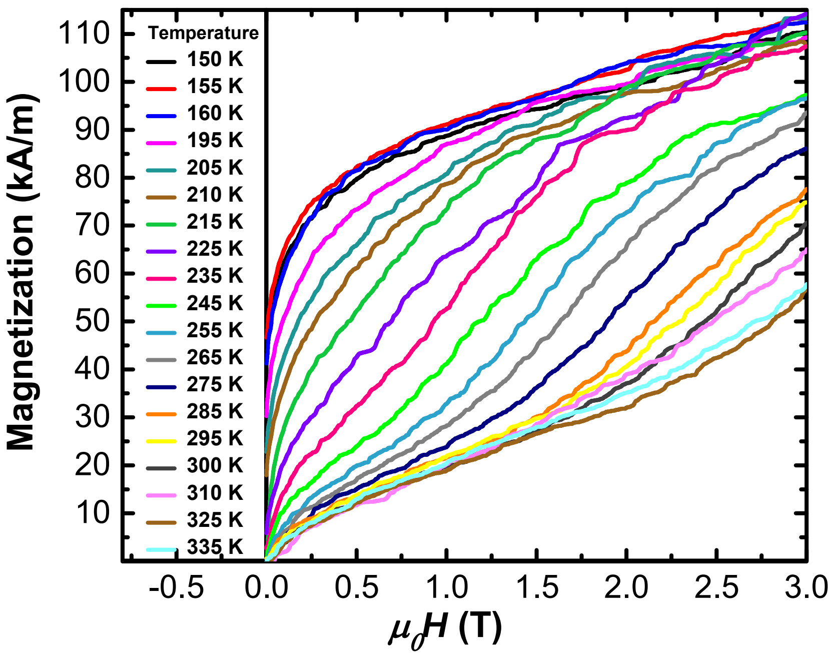

Figure S6. Magnetization isotherms measured by sweeping the magnetic field from 0 to 3 T at different temperatures between 150 K and 340 K, i. e. across the first order magnetic phase transition in the Mn_2GaC film.
